# Understanding the Role of Nitronate Monooxygenases in Virulence of the Human Fungal Pathogen *Aspergillus fumigatus*

**DOI:** 10.3390/jof8070736

**Published:** 2022-07-16

**Authors:** Phuong Tuyen Nguyen, Theresa Wacker, Alistair J. P. Brown, Alessandra da Silva Dantas, Elena Shekhova

**Affiliations:** MRC Centre for Medical Mycology, University of Exeter, Geoffrey Pope Building, Stocker Road, Exeter EX4 4QD, UK; nguyenptuyen@gmail.com (P.T.N.); tw492@exeter.ac.uk (T.W.); a.j.p.brown@exeter.ac.uk (A.J.P.B.); a.da-silva-dantas@exeter.ac.uk (A.d.S.D.)

**Keywords:** redox biology, *Aspergillus fumigatus*, macrophages, peroxynitrite, nitronate monooxygenases

## Abstract

*Aspergillus fumigatus* is the leading cause of the fungal invasive disease called aspergillosis, which is associated with a high mortality rate that can reach 50% in some groups of immunocompromised individuals. The increasing prevalence of azole-resistant *A. fumigatus* isolates, both in clinical settings and the environment, highlights the importance of discovering new fungal virulence factors that can potentially become targets for novel antifungals. Nitronate monooxygenases (Nmos) represent potential targets for antifungal compounds as no orthologs of those enzymes are present in humans. Nmos catalyse the denitrification of nitroalkanes, thereby detoxifying these mediators of nitro-oxidative stress, and therefore we tested whether Nmos provide protection for *A. fumigatus* against host-imposed stresses at sites of infection. The results of inhibition zone assays indicated that Nmo2 and Nmo5 are not essential for the oxidative stress resistance of *A. fumigatus* in vitro. In addition, the resazurin-based metabolic activity assay revealed that the growth of mutants lacking the *nmo2* or *nmo5* genes was only slightly reduced in the presence of 0.05 mM peroxynitrite. Nevertheless, both Nmo2 and Nmo5 were shown to contribute to defense against murine bone marrow-derived macrophages, and this was no longer observed when NADPH oxidase, the main generator of reactive oxygen species during infection, was inhibited in macrophages. Furthermore, we revealed that Nnmos promote the virulence of the fungus in the *Galleria mellonella* model of infection. Both *nmo2* and *nmo5* knock-out strains were less virulent than the wild-type control as recorded 72 h post-infection. Our results indicate that Nmos play a role in the virulence of *A. fumigatus*.

## 1. Introduction

Fungal infections affect hundreds of millions of people globally each year [1]. Diseases caused by pathogenic fungi lead to approximately 1.7 million deaths annually, which is roughly equivalent to tuberculosis, which causes about 1.5 million deaths per year [1,2].

*Aspergillus fumigatus* is one of the major fungal pathogens of humans. Individuals with a weakened immune system are especially at risk of morbidity and mortality from *A. fumigatus*-associated infections [3]. The mortality rate of invasive aspergillosis in patients with leukaemia or those undergoing hematopoietic stem cell transplantation can reach as high as 50% [4,5]. Furthermore, antifungal resistant *A. fumigatus* strains are emerging and on the rise [6,7]. These strains appear to be derived from the use of antifungal compounds in agriculture that are driving this emergence of azole resistance in the environmental [8,9].

One of the ways to combat the emergence of antifungal resistance is to develop new antifungal therapies. Investigations of host–pathogen interactions may help us to identify essential fungal virulence factors that may represent novel targets for antifungal compounds. Immune cells possess various natural mechanisms to kill invading pathogens, including the production of reactive oxygen species (ROS) and reactive nitrogen species (RNS), which together can create nitro-oxidative stress [10]. However, *A. fumigatus* has defensive mechanisms that reduce damage caused by nitro-oxidative stress, thereby maintaining normal growth under such conditions [11]. Therefore, exploring the mechanisms by which *A. fumigatus* copes with nitro-oxidative stress will increase our understanding of how this fungus mitigates host-imposed stresses and thus causes infections.

In a recent study it was discovered that nitronate monooxygenase (Nmo) in the filamentous fungus *Magnaporthe oryzae* is necessary to resist nitro-oxidative stress stimulated during colonisation of its plant host [12]. The authors reported that this fungus employs a specific Nmo protein, Nmo2, to convert nitro-oxidative-stress-induced toxic compounds (nitroalkanes) into other nitrogen sources that might promote fungal proliferation. Intriguingly, the *M. oryzae NMO2* gene had a dramatic effect on host redox homeostasis, subsequently facilitating pathogenic colonisation and the establishment of rice blast disease.

There are five potential Nmo-encoding genes in *A. fumigatus*. Interestingly, no orthologs are found in humans, indicating that these enzymes might be potential antifungal targets. We reasoned that, similarly to *M. oryzae* Nmo2, Nmo2 may play a significant role in the virulence of *A. fumigatus*. We hypothesised that Nmo2 might protect the fungus against oxidative and nitro-oxidative stresses during interactions with immune cells such as macrophages. We were also interested in characterising Nmo5, which shares 57% amino acid sequence similarity to Nmo2. Therefore, we have compared the biological activities and phenotypes of *A. fumigatus* wild-type, Δ*nmo2*, and Δ*nmo5* strains. Our data show that Nmo2 and Nmo5 have no notable effect on redox balance in either fungal or host cells in vitro, but that these enzymes do contribute to the ability of *A. fumigatus* to cause infection.

## 2. Materials and Methods

### 2.1. Generation of Mutants

*A. fumigatus* CEA10 was used as a parental strain and is referred to here as the wild-type strain. To generate Δ*nmo2* and Δ*nmo5* deletion strains, a strategy involving homologous recombination, followed by the transformation of protoplasts [13] (Figure S1). Targeted replacement cassettes were obtained by amplifying both flanking regions from the genomic DNA and fusing them with pyrithiamine resistance marker of pSK275 plasmid (a generous gift from Prof. Sven Krappmann) [14]. A Gibson assembly, using NEBuilder^®^ HiFi DNA Assembly Master Mix (New England Biolabs, Frankfurt, Germany), was used to create constructs. The used primers are listed in Table S1. The correct integration was confirmed by means of PCR using the primers listed in Table S2.

### 2.2. Cultivation of A. fumigatus Strains

*Aspergillus* minimal medium (AMM) agar media, described in a previous study with several modifications, was used [15,16]. The media contained 1.5% purified agar (Oxoid, Basingstoke, UK), 10xSalt solution, 20% Glucose, 20% MgSO_4_, trace elements, and water. This medium had glucose as the main carbon source, and sodium nitrate was added as a source of nitrogen. After five days of incubation at 37 °C, conidia were harvested in sterile water, and filtered through a 40-μm cell strainer (BD Biosciences, Heidelberg, Germany). Following vigorous vortexing, the concentration of conidia was determined using a Vi-CELL analyser (Beckman Coulter, Brea, CA, USA) or a hemocytometer.

Swollen conidia of *A. fumigatus* were obtained following the method described in the aforementioned published article [17]. Conidia were grown in liquid Sabouraud dextrose media for 3 h with shaking at 30 rpm. After swelling, the swollen conidia were washed and then re-suspended in RPMI 1640 Medium, GlutaMAX 25 mM HEPES media, and 10% FBS (R10 medium).

### 2.3. Nitronate Monooxygenase Enzymatic Activity Assay

All strains were grown in AMM liquid medium at the concentration of 10^6^ conidia/mL. These cultures were incubated at an optimal growth temperature of 37 °C, with shaking at 180–200 rpm for more than 24 h. The fungal mycelium was harvested, washed with sterilised water, dried with paper, and ground using liquid nitrogen, mortar, and pestle. Phosphate-buffered saline (PBS) containing a protease inhibitor cocktail was added to the cell biomass, and it was subsequently incubated for 10 min on ice. During this time, samples were vortexed several times to enhance the amount of protein extracted. Samples were centrifuged for 5 min at 13,000 rpm and the supernatant was transferred to new tubes.

The Bradford assay was conducted to quantify the exact total amount of protein in each sample. After that, the enzymatic mix was prepared, which included 50 µM 2-nitropropane (Sigma-Aldrich, St. Louis, MO, USA), PBS, and a sample supernatant with a protein concentration between 100–200 µg. The enzymatic mix was then gently shaken and incubated at 37 °C. After that, 10 µL of concentrated HCl was added and the mix was placed on ice for 5 min. This was required for the formation of HNO_2_. The reaction mix was prepared in a ratio of 1:1, which contained 1% sulfanilamide (Sigma-Aldrich) in 3N HCl and 0.02% N-(l-naphthyl) ethylenediamine dihydrochloride (Sigma-Aldrich) in water.

The reaction mix was added to a 96-well plate, followed by the addition of equal amount of the enzymatic mix. The absorbance colour was read at 540 nm every minute for 20 min in total. The control used was a mixture of reaction mix and water. Enzymatic activity was expressed as a change in absorbance per mg of total protein.

### 2.4. Oxidative Stress Sensitivity Assays

To perform the inhibition zone assay, Petri dishes with 10 mL AMM bottom-agar were overlayed with 10 mL AMM top-agar containing conidia at the concentration of 10^7^. The conidia suspension was added to AMM top-agar before it solidified. After agar solidification, holes were punched in the centre of the culture plates and 100 µL of specific oxidative stressors were subsequently added. The concentrations of particular stressors were as follows: 128 mM tert-butyl peroxide, 3% H_2_O_2_, 12.5 mM menadione, and 0.1 M diamide. Inhibition zones were measured in diameter (cm) after 24 h of incubation at 37 °C.

In the droplet assay, 0.1 mM tert-butyl peroxide was added to AMM agar plates. The concentration of conidia suspension was 2 × 10^5^ spores/mL and 5 µL of the suspension was spotted in the middle. The growth zones were observed every 24 h for 4 days. The control used was AMM agar plates.

### 2.5. Nitro-Oxidative Stress Assay

Peroxynitrite (Sigma, 516620-1SET) at the final concentration of 0.05 mM was prepared in AMM and distributed to the 96-well plates. Fungal spores at the concentration of 2 × 10^6^ conidia/mL were prepared in AMM liquid media containing 0.004% of resazurin. Measurements were carried out during incubation for 24 h at 37 °C. Results were obtained using the TECAN system, and the percentage of fungal cell survival was calculated.

### 2.6. BMDM Killing Assays, Cytokine Secretion, and Protein Abundance Measurements

Murine bone marrow-derived macrophages (BMDMs) from C57BL/6J wild-type mice were differentiated, following a previously described protocol [17,18]. All animal use was in compliance with the animal research ethical regulations and a UK Home Office project licence number P79B6F297. Cellular differentiation analysis was conducted using RPMI 1640, GlutaMAX 25 mM medium supplemented with 20% L929 cell supernatant, 10% foetal bovine serum (FBS), 1% penicillin, and 1% streptomycin. Cells were incubated at 37 °C and 5% CO_2_ over 7 days with 2 instances of new media exchange. One day before experiments, BMDMs were harvested and transferred into R10 media.

BMDMs in R10 medium were seeded into the tissue culture-treated surface 96-well plates at 0.5 × 10^6^ cells/well and left to adhere overnight. Resting conidia (killing assay) or swollen conidia (cytokine measurement) were added to cells with MOI 1 or 3, respectively, and incubated at 37 °C for 16–20 h. For the killing assay, macrophages were lysed with 0.1% Triton solution and resazurin was added. Measurements were carried out using the TECAN plate reader. To measure cytokine secretion, supernatants from macrophages were collected at 14 h (overnight) after stimulation with swollen conidia. IL-10 (DY417), IL-1β (DY401), and TNF-α (Dy410) ELISA kits from R&D Systems (Minneapolis, MN, USA) were used according to the manufacturer’s instructions. BMDMs were also incubated at 37 °C for 2 h following infection with swollen conidia. Cell homogenates were then used to assess the abundance of proteins in infected BMDMs. Extracted proteins were analysed via western blotting with anti-GLRX1 (R&D Systems, AF3119), anti-SOD2 (Abcam, Cambridge, UK, ab13533), NOX2 (Abcam, ab129068), or anti-GAPDH (Abcam, ab181602) antibodies. For independent experiments, new batches of conidia and macrophages were used.

### 2.7. In-Gel Redox Proteomics

The enrichment of reversibly oxidised proteins from infected BMDMs was performed as previously described [19]. Briefly, proteins from BMDMs were precipitated with TCA, whereas proteins containing free thiols were blocked with iodoacetamide. Reversibly oxidised proteins were reduced using dithiothreitol and enriched on thiopropyl Sepharose 6B resin. Eluted proteins were visualised using silver-stained SDS gels.

### 2.8. Galleria mellonella Infection Model

Larvae of *G. mellonella* that were used in the experiment had a uniform colour, normal mobility, and weights between 150 and 350 mg. Larvae were chosen randomly to allocate them into six groups (12 larvae per group), which included the control group. The spore suspensions of the three *A. fumigatus* strains were prepared in PBS with 2 concentrations (10^5^ and 10^6^ conidia/mL). Each larvae group was then injected with 30 µL per larvae at the last proleg using syringes. The larvae in the control group were injected with PBS. Incubation took place at 37 °C, and mortality rates were recorded every 24 h for 3 days. Based on the result obtained, the mortality of the larvae from each strain was calculated.

### 2.9. Statistical Analysis

The statistical testing was performed using GraphPad Prism. These tests included the one-way ANOVA test, two-way ANOVA test, Kaplan–Meier survival analysis (Log-rank test), and Tukey’s multiple comparison test.

## 3. Results

### 3.1. Deletion of Nmo2 and Nmo5 Genes Did Not Significantly Impact the Short-Term Response of A. fumigatus to Oxidative Stress

Our initial aim was to investigate the biological activities of *A. fumigatus* Nmo2, as its homolog in the plant fungal pathogen *M. oryzae*, Nmo2 (MGG_02439), was shown to be important for virulence. In order to identify conserved amino acid sequences in Nmo2 (AFUA_4G07940, accession number XP_752031.1), we performed a BLAST analysis using non-redundant protein sequences in *A. fumigatus* (taxid:330879). Similarly to *M. oryzae*, we found four other proteins corresponding to Nmoss: Nmo1 (AFUA_7G03850, accession number XP_749123.1), Nmo3 (AFUA_2G17430, accession number XP_756078.1), Nmo4 (AFUA_5G09600, accession number XP_753679.1), and Nmo5 (AFUA_2G09850, accession number XP_755318.1). Here, BLAST analysis showed that Nmo2 and Nmo5 share 57% sequence identity, whereas Nmo1, Nmo3, and Nmo4 share less than 40% sequence identity with Nmo2. Moreover, multiple protein sequence alignment of the *A. fumigatus* Nmos using Clustal Omega (version 1.2.4) confirmed that Nmo2 and Nmo5 possessed similar amino acid sequences (Figure S2). Therefore, for our further functional analysis, we investigated Nmo5 along with Nmo2. Although those two proteins possess similar amino acid sequences, our computational prediction showed that they might have different cellular localisations (Table S3). This suggested that their biological functions might differ.

Furthermore, to validate that *A. fumigatus* Nmo2 and Nmo5 have all the necessary functional domains for their activity, sequences of *A. fumigatus*, *M. oryzae*, and *M. brunneum* Nmos were used as queries for an rpsblast search of the Conserved Domain Database. Sequences of *M. oryzae* and *M. brunneum* were chosen here for the comparison as Nmos in those fungi have been proven to be functional [12,20]. Furthermore, Interpro Scan software was utilised to scan for domains and to visualise them. Both approaches revealed that all the domains necessary for Nmo2 and Nmo5 to function were present (Table S4, Figure S3). Finally, we compared the sequence identity of Nmos in those three fungal pathogens (Tables S5 and S6, Figure S4). This provided additional evidence about the similarity of Nmo2 and Nmo5 in those organisms.

**Figure 1:**
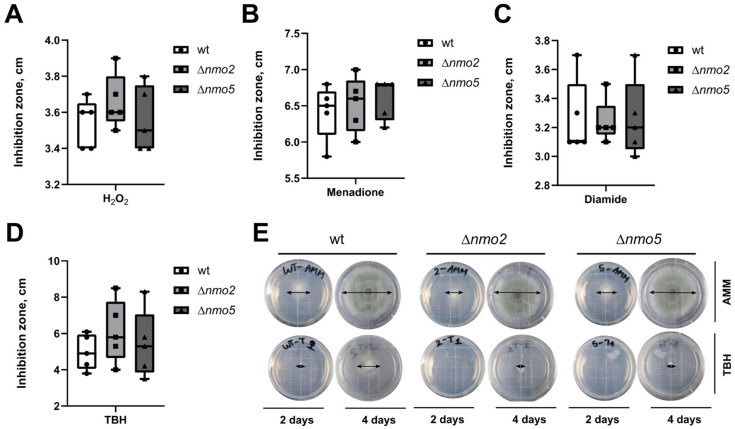
Phenotypic characterisation of wild-type, Δ*nmo2,* and Δ*nmo5 A. fumigatus* strains after being treated with various oxidative stressors. The sensitivity of *A. fumigatus* strains to different oxidative stress-inducing compounds was evaluated using inhibition zone (**A**–**D**) and droplet (**E**) assays. The inhibition zones in the diffusion assay were measured after adding a specific oxidative compound—(**A**) H_2_O_2_, (**B**) menadione, (**C**) diamide, and (**D**) tert-butyl peroxide (TBH). Circular-shaped data points correspond to wild-type, square-shaped to Δ*nmo2*, and triangular-shaped *to* Δ*nmo5*. In the droplet assay (**E**), 0.1 mM tert-butyl peroxide was added to agar plates. Black arrows indicate the diameters of fungal colonies. Data were obtained from 5 independent experiments, and the statistical test used was the one-way ANOVA test.

To study the importance of Nmo2 and Nmo5, three different *A. fumigatus* strains were used: the wild-type parental strain CEA10 (CBS144.89), a Δ*nmo2* null mutant, and a Δ*nmo5* null mutant (Table S7). First, we tested whether these Nmos promoted nitro-oxidative stress resistance in *A. fumigatus*. It is well established that peroxynitrite contributes to the creation of nitro-oxidative stress and is formed upon reaction between ROS such as superoxide and nitric oxide (NO) [21]. NO is normally generated as a by-product of nitrogen metabolism during the utilisation of NO_3_^−^ and NO_2_^−^ and is thus always present in fungal cells grown on these nitrogen sources [22]. Therefore, we first tested how the deletion of *nmo2* and *nmo5* affected the growth of the fungus on NO_3_^−^-containing media with ROS-generating compounds. Here, inhibition zone assays were employed (Figure 1A–D). The inhibition zones (measured in centimetres) were recorded after 24 h of incubation and they showed that hydrogen peroxide and tert-butyl hydroperoxide inhibited the growth of the deletion strain Δ*nmo2*, but trends were not statistically significant. Δ*nmo2* was more inhibited by tert-butyl hydroperoxide than the wild-type. Therefore, we further performed another type of assay testing the sensitivity to this compound. Here, a droplet assay showed that unlike the wild-type control, both Δ*nmo2* and Δ*nmo5* mutants showed almost no growth when exposed to tert-butyl hydroperoxide for four days (Figure 1E). In conclusion, Nmo2 and Nmo5 appear to be important for long-term resistance to tert-butyl hydroperoxide, but not for the short-term stress response to this compound.

### 3.2. Nmo2 and Nmo5 Promote Resistance to Some Nitro-Oxidative Stresses In Vitro and Are Not Required for Enzymatic Detoxification of Nitropropane

**Figure 2:**
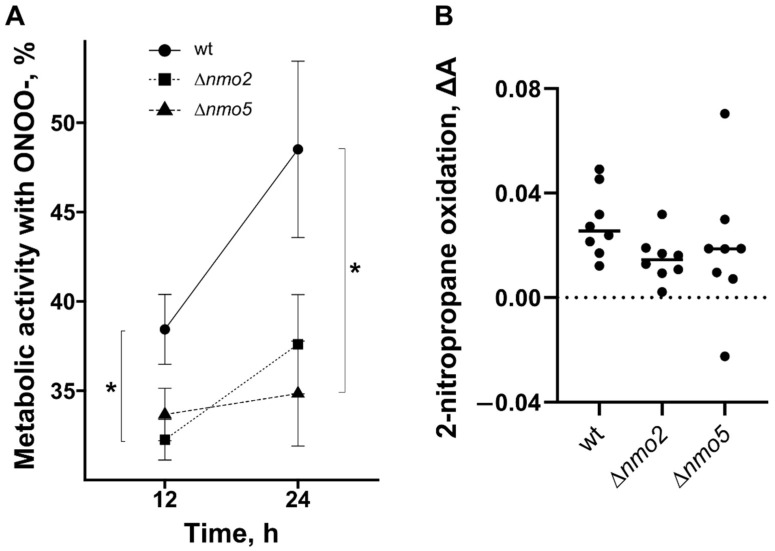
Evaluation of the importance of Nmo2 and Nmo5 for protection against peroxynitrite and the enzymatic denitrification of nitropropane. (**A**) Percentage of metabolic activity of *A. fumigatus* strains after being treated with peroxynitrite at a concentration of 0.05 mM. Data were obtained from 2 independent experiments with 16 replicates in each group. The statistical test used was a one-way ANOVA test; * indicates *p* < 0.05. (**B**) Determination of enzymatic activity levels using proteins isolated from *A. fumigatus* strains in the presence of a specific substrate—nitropropane. The amount of accumulated product was measured spectroscopically as a change in absorbance, which was normalised to the total amount of protein in a reaction mix. Data were obtained from 4 independent experiments.

Next, we tested the impact of the deletions upon nitro-oxidative stress resistance in vitro (Figure 2A). Resazurin, which was proven to be a reliable indicator of *A. fumigatus* viability [23], was added as a dye/indicator of the metabolic activity of the fungus in the presence of peroxynitrite. In this assay, compared to the wild type, Δ*nmo2* showed reduced metabolic activity after 12 h of incubation with 0.05 mM of peroxynitrite, whereas the metabolic activity of Δ*nmo5* was significantly lower after 24 h of incubation (Figure 2A). Notably, peroxynitite was highly unstable and was quickly decomposed during storage [24], leading to the loss of its ability to inhibit cellular growth.

Nmos protect cells from nitro-oxidative stress by catalysing the oxidative denitrification of toxic nitroalkanes such as 2-nitropropane. To test whether the lack of Nmos results in reduced ability of the fungus to denitrify nitroalkanes, we performed an enzymatic activity assay to evaluate enzymatic 2-nitropropane oxidation by proteins isolated from each strain. Total proteins were extracted from the cell biomass to perform this assay, in which 2-nitropropane (nitroalkane) was used as a substrate for the reaction. According to the data (Figure 2B), both Δ*nmo2* and Δ*nmo5* strains showed a slight decrease in enzymatic activity. However, there was no significant difference when results from several experiments were compared. It is likely that changes in enzymatic activity were not observed in the deletion strains, because the applied substrate (nitropropane) was not converted specifically by Nmo2 or Nmo5, but rather by other remaining nitronate monooxygenases in these mutants. To sum up, Nmo2 and Nmo5 might contribute to peroxynitrite resistance in vitro (Figure 2A), which is independent of the enzymatic oxidation of 2-nitropropane.

### 3.3. Nmo2 and Nmo5 Promote Resistance to Macrophage and Galleria mellonella Killing

**Figure 3:**
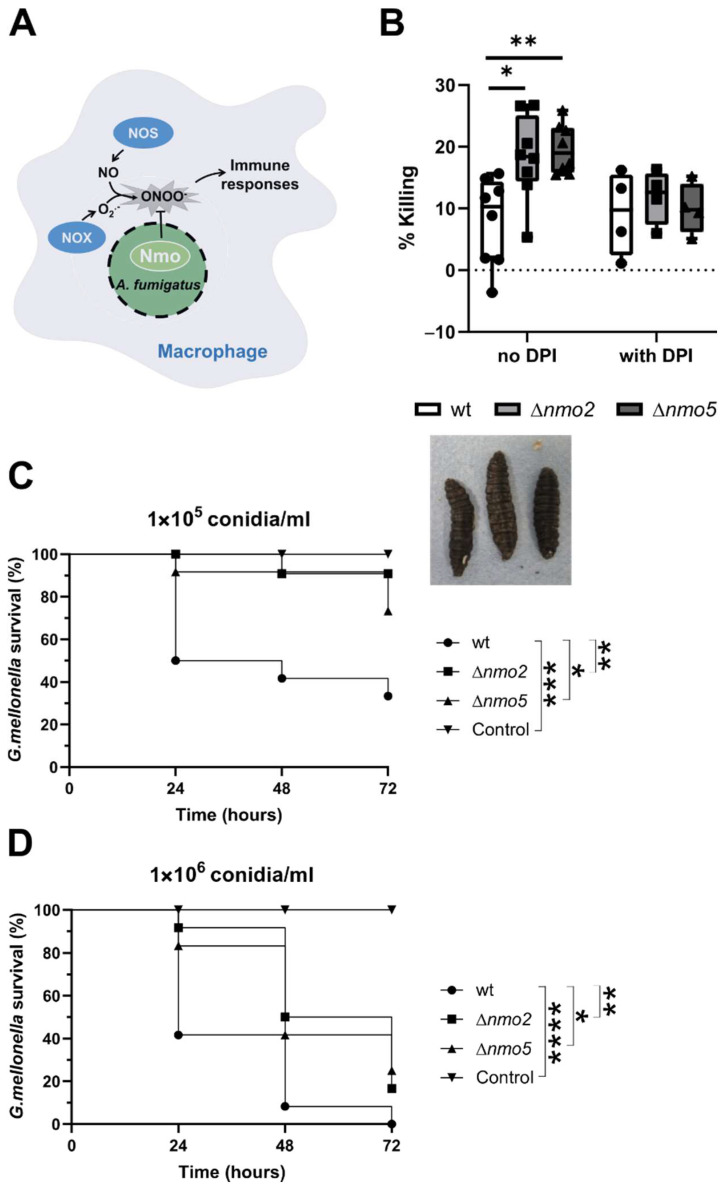
Investigation of the virulence of *A. fumigatus* Δ*nmo2* and Δ*nmo5*. (**A**) Nitro-oxidative homeostasis is altered in infected phagocytes. Macrophages produce nitric oxide that reacts with NADPH-oxidase derived superoxide to form a highly reactive nitrogen intermediate, peroxynitrite. Peroxynitrate acts as signalling molecules and thus can affect immune responses. Nmos, in turn, can either directly or indirectly mitigate biological activities of peroxynitrite. (**B**) Percentage of fungal killing by BMDMs either with or without DPI pre-treatment. Data were obtained from 3 independent experiments. Circular-shaped data points correspond to wild-type, square-shaped to Δ*nmo2*, and triangular-shaped *to* Δ*nmo5*. The statistical test used was the two-way ANOVA test; * indicates *p* < 0.05 and ** indicates *p* < 0.01. (**C**,**D**) Kaplan–Meier survival curves of *Galleria mellonella* larvae infected with wild-type, Δ*nmo2*, and Δ*nmo5 A. fumigatus* strains. Larvae were infected with (**A**) 1 × 10^5^ conidia/mL or (**B**) 1 × 10^6^ conidia/mL of *A. fumigatus*. The control group were injected with a 30 μL dose of PBS solution. Surveillance was conducted every 24 h over a period of 72 h. Twelve biological replicates were included in each group. The statistical test used was the Log-rank test; * indicates *p* < 0.05, ** *p* < 0.01, *** *p* < 0.001, and **** *p* < 0.0001, respectively.

It is well-established that nitro-oxidative homeostasis is shifted in infected phagocytes. Macrophages produce nitric oxide (NO) that, during phagocytosis, can react with NADPH oxidase-derived superoxide to form peroxynitrite (Figure 3A). In addition to its direct antimicrobial activities, peroxynitrate acts as a signalling messenger due to its ability to modify protein structures and functions, and thus can influence regulatory processes that control immune responses [25]. We therefore postulated that Nmos could have an impact on immune responses and affect the ability of cells to clear fungal conidia efficiently (Figure 3A). This was supported by our observation that Δ*nmo2* and Δn*mo5* strains were better eliminated by murine bone marrow-derived macrophages (Figure 3B). Interestingly, this difference in killing was no longer observed when immune cells were pre-treated with diphenylene-iodonium chloride, an inhibitor of NADPH oxidase [26] that decreases the ability of macrophages to generate nitro-oxidative stress (Figure 3B). This suggested that *A. fumigatus* Nmo2 and Nmo5 attenuate NADPH oxidase-dependent immune responses of macrophages against the fungus.

To further confirm the importance of Nmos for the virulence of *A. fumigatus*, we utilised an in vivo insect model (*Galleria mellonella*) of infection. Here, larvae were randomly distributed into six groups and were inoculated with conidia suspensions at two different concentrations. After infection, the mortality rates of the host were recorded every 24 h via visible factors such as mobility, the lack of silk cocoon formation, and the extent of melanisation (Figure 3C,D). Using different concentrations of conidia, we could show that after 72 h of incubation, larvae infected with deletion strains showed a significantly higher survival rate than wild-type-infected insects (Figure 3C,D). Thus, we concluded that Nmo2 and Nmo5 play a role in virulence of *A. fumigatus*.

### 3.4. Nmo2 and Nmo5 Do Not Affect Macrophage Redox Homeostasis or Cytokine Secretion

**Figure 4:**
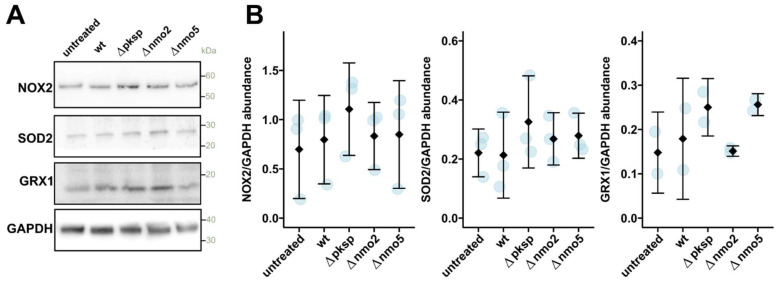
Examination of redox homeostasis in macrophages during infection. (**A**) Western blot analysis of protein abundance in BMDMs untreated or infected for 2 h with swollen conidia. Proteins were isolated from macrophages and evaluated via western blot with NOX2 (anti-gp91phox), SOD2, GRX1, or GAPDH antibodies. (**B**) Quantification of the intensity of corresponding bands on blots as shown on (**A**) from 3 (NOX2, SOD2) and 2 (GRX1) independent experiments. Images were processed in ImageJ. The integrated density of each band was measured after background subtraction and image inversion. Values were normalised to the integrated density from the corresponding lanes. Data shown in relation to a band intensity for GAPDH obtained in each experiment. Rhombs and error bars indicate means and standard deviation.

We next tested whether Nmos can alter nitro-oxidative homeostasis in immune cells. In the case of disrupted nitro-oxidative homeostasis, macrophages might react by increasing the abundance of antioxidant proteins while reducing the abundance of superoxide-producing NADPH oxidase. Interestingly, our study showed that when macrophages were infected with tested strains, there were no significant changes in the abundance of NADPH oxidase or major host antioxidant enzymes, namely, superoxide dismutase 2 and glutaredoxin 1 (Figure 4A,B). Here, we also included the Δ*pksP* strain in our analysis, which is known to be able to cause elevated ROS production in immune cells [27], and we used this as a positive control. Indeed, there was a slight increase in the abundance of antioxidant enzymes in macrophages infected with Δ*pksP* compared to the wild-type strain; however, this difference was not statistically significant (Figure 4A,B). To further evaluate the cellular redox state, we performed an in-gel analysis of enriched reversibly oxidised proteins in infected macrophages (Figure S5A). In this assay, oxidised proteins were enriched and loaded onto the gel for visualisation. Interestingly, we could not observe a consistent difference in the redox state of proteins in macrophages infected with wild-type vs. Δ*nmo2* strains (Figure S5B,C). Considering these findings together, Nmo2 does not significantly alter the abundance of antioxidant enzymes or the redox state of proteins in infected macrophages.

**Figure 5:**
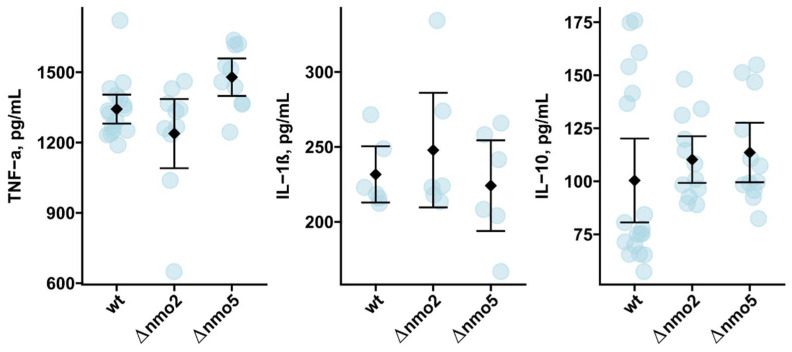
Assessment of cytokine secretion by bone-marrow-derived macrophages during infection with *A. fumigatus*, Δ*nmo2*, and Δ*nmo5*. BMDMs were exposed to swollen *A. fumigatus* conidia and the supernatant was collected after incubation overnight. Levels of TNF-α, IL-1β, and IL-10 in supernatants were analysed via ELISA. Data were obtained from 2 independent experiments. Rhombs and error bars indicate means and standard deviation.

Finally, we tested whether Nmo2 and Nmo5 might impact the macrophage production of inflammatory mediators—cytokines. Our focus was on TNF-α, IL-1β, and IL-10, which are among key regulators of antifungal immune responses and fungus-induced inflammation [28]. Here, we observed that the production of inflammatory TNF-α and IL-1β and anti-inflammatory IL-10 was not changed following infection with Δ*nmo2* and Δn*mo5* in contrast to infection with the wild-type strain (Figure 5). Therefore, Nmo2 and Nmo5 contribute to the protection of *A. fumigatus* from killing by macrophages (Figure 3), but this cannot be explained by their effects on cytokine secretion by macrophages.

## 4. Discussion

Nmos can detoxify harmful compounds (nitroalkanes) that accumulate in cells during nitro-oxidative stress [12,29,30,31,32]. However, few studies have been conducted to characterise their functional roles in living organisms such as fungi [12,20]. Recently, the importance of Nmos for fungal virulence has been demonstrated in *M. oryzae* and *Metarhizium brunneum* during rice and insect infections, respectively [12,20]. As far as we know, our study represents the first attempt to understand the roles of specific Nmos in the human fungal pathogen *A. fumigatus*.

Our results showed that Nmo2 and Nmo5 are not required for the short-term oxidative stress resistance of *A. fumigatus* in vitro and might only be necessary for long-term responses. In contrast, a study by Marroquin-Guzman et al. revealed that *M. oryzae* Δ*nmo2* exhibited apparent growth impairment on culture agar plates with hydrogen peroxide [12]. In our research, compared to the wild type, Δ*nmo2* and Δ*nmo5* were not more inhibited by hydrogen peroxide but displayed reduced growth after prolonged incubation with a specific oxidative-stress-inducing tert-butyl peroxide. It is likely that such a difference can be explained by the presence of three catalases in *A. fumigatus*, which provide a protection against immediate oxidative stress [33]. In contrast, only two heme catalases were found in the genome of *M. oryzae* [34,35]. Given that tert-butyl peroxide is a more stable and potent alkyl hydroperoxide than hydrogen peroxide [36], the prolonged exposure to this compound led to oxidative and subsequently nitro-oxidative stresses. Therefore, Nmos might act in concert with catalases to protect *A. fumigatus* from stresses.

Our research also revealed that Nmos contribute to the protection of *A. fumigatus* from macrophages. This cannot be solely explained by the involvement of these enzymes to stress response systems. This is because Δ*nmo2* and Δ*nmo5* have only minor sensitivity to oxidative and nitro-oxidative stresses. Previously, deletion strains such as Δ*Afyap1* and Δ*Afskn7* with much more dramatic sensitivity to oxidative stress showed no importance in relation to the protection from killing by immune cells [37,38]. Another explanation can be that *A. fumigatus* Nmos, similarly to Nmo2 in the plant pathogen *M. oryzae*, could impact the redox homeostasis of host cells and thus alter their immune responses. However, we could not observe any detectable effect of Nmos on the production of macrophage antioxidative enzymes following infection with *A. fumigatus*. Another possibility would be that infection-associated peroxynitrte could induce the nitrosylation of various regulatory proteins of immune cells and Nmos could indirectly affect those regulators by interacting with host peroxynitrite. Indeed, in immune cells, protein nitrosylation may promote immunosuppression by exhibiting inhibitory effects on cellular immunomodulators such as NF-kβ transcription factor and mitogen-activated protein kinases [39,40]. However, as measured by the secretion of cytokines, which are in part regulated by NF-kβ and mitogen-activated protein kinases, Nmo2 and Nmo5 did not affect those immunomodulators in macrophages. Thus, more research is needed to provide mechanistic insights into the importance of Nmos for the protection of *A. fumigatus* from attack by immune cells.

Finally, we have provided additional evidence that Nmo2 and Nmo5 can be important in establishing fungal infection as both Δ*nmo2* and Δ*nmo5* displayed impaired virulence in an in vivo insect *Galleria mellonella* model. In corroboration with the fact that proteins homologous to Nmos are absent in humans, this supports our idea that Nmos may prove themselves as possible targets for antifungals to treat invasive aspergillosis. Intriguingly, a previous study showed that orthologs of Nmos are also found in pathogenic bacteria such as *Mycobacterium tuberculosis* and *Pseudomonas aeruginosa*. It was proposed that these enzymes might be responsible for the fitness attributes of the bacteria during infection [31,41]. Furthermore, some progress has been made in finding small molecules that can inhibit Nmos’ functions in *M. tuberculosis* and thus become useful in treatment of tuberculosis [42]. Therefore, further investigations of Nmos might be potentially translated into clinical applications to treat the microbial co-infections simultaneously.

## 5. Conclusions and Perspectives

In our study, the importance of *A. fumigatus* Nmo2 and Nmo5 for virulence was shown using in vitro macrophage and in vivo *G. mellonella* infection models. Although *G. mellonella* has been proven as a useful model to study fungal virulence, its drawbacks include evolutionary genetic divergence between mammals and insects, and the lack of an equivalent to the mammalian adaptive immune response [43]. In future studies, virulence assessments should, therefore, be carried out with different models, such as murine models. Our research also indicates that *A. fumigatus* nitro-oxidative stress responses are beneficial against host immunity. To gain more insights into *A. fumigatus*–host interactions, further investigations are needed to reveal the exact molecular mechanisms by which nitro-oxidative stress responses are activated and regulated in *A. fumigatus* during infection. Finally, to further evaluate the potential of Nmos to become targets for antifungals, the generation of recombinant proteins will be useful to fully understand the biochemical properties of these enzymes, and to predict and design new molecules for their inhibition.

## Data Availability

Not applicable.

## Supplementary Materials

The following supporting information can be downloaded at https://www.mdpi.com/article/10.3390/jof8070736/s1, Table S1: Primers used in this study to generate *A. fumigatus* mutant strains; Table S2: Primers used for validation of deletion strains; Table S3: Signal peptide and cellular localisation predictions for *A. fumigatus* Nmos; Table S4: Conserved protein domains in Nmo2 and Nmo5 of *Magnaporthe oryzae*, *Metarhizium brunneum*, and *Aspergillus fumgatus*; Table S5: Global similarity (Blosum62) results of MAFFT alignment; Table S6: Identity results of MAFFT alignment; Table S7: *Aspergillus fumigatus* strains used in this study; Figure S1: Validation of *nmo2* and *nmo5* gene deletion; Figure S2: Protein sequence analysis of Nmos in *A. fumigatus*; Figure S3: Interpro Scan results for Nmo2 and Nmo5 of *Aspergillus fumigatus* and *Magnaporthe oryzae*; Figure S4: Maximum likelihood tree of Nmo1–5 in *Aspergillus fumigatus*, *Magnaporthe oryzae*, and *Metarhizium brunneum*; Figure S5: Protein oxidation in infected BMDMs [44,45,46,47,48,49,50,51,52,53,54,55].
