# Supplementary material for: Understanding the Role of Nitronate Monooxygenases in Virulence of the Human Fungal Pathogen Aspergillus fumigatus
Source: J Fungi (Basel). 2022 Jul 16;8(7):736. doi: 10.3390/jof8070736 (PMC9323177; doi:10.3390/jof8070736)
Supplement: Supplementary file 1 [file jof-08-00736-s001.zip › jof-1788152-SI.pdf]

Supplemental materials

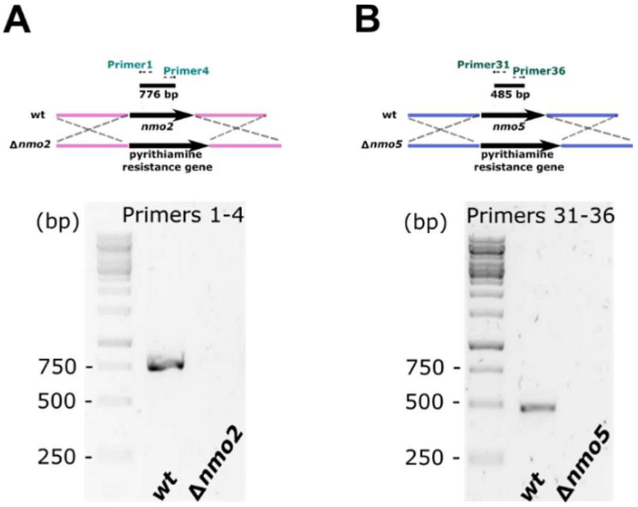

Supplemental Figure S1. Validation of *nmo2* and *nmo5* gene deletion.

The PCR was done using genomic DNA isolated from cultivated strains. Set of pair-primers used: A. Primer 1-4: to detect *nmo2* gene presence. B. Primer 31-36: to detect *nmo5* gene presence.

Name	Sequence 5' - 3'
9_ES_LF_4g07940_pSK275_fw	AGGAATTCGATGGCCACTGTGGAGGATACACTCAATAACAG
10_ES_LF_4g07940_pSK275_rev	TGGGATCCCGTAATCAATTGTCGTTTAGCTGTATGTTCCG
11_ES_PtrA_LF_4g07940_fw	CGGAACATACAGCTAAACGACAATTGATTACGGGATCCCA
12_ES_PtrA_RF_4g07940_rev	GCCTAGTCCATTATCTAAGCTCAATTCTAGAATGCCCCACCG
13_ES_RF_4g07940_PtrA_fw	CGGTGGGGCATTCTAGAATTGAGCTTAGATAATGGACTAGGC
14_ES_RF_4g07940_pSK275_rev	GATGGCCTCTTGCATCTTGACAACAGCCTTCGGG
24_ES_LF_2g17430_pSK275_fw	CAGGAATTCGATGGCCACTGTTGGAACCGGCTGATACAT
25_ES_LF_2g17430_pSK275_rev	TGGGATCCCGTAATCAATTGGGACCATGGCCTTAAGACTG
26_ES_PtrA_LF_2g17420_fw	CAGTCTTAAGGCCATGGTCCCAATTGATTACGGGATCCCA
27_ES_PtrA_RF_2g17420_rev	CTGCGCAAACCACCAAACAAGATTCTAGAATGCCCCACCG
28_ES_RF_2g17430_PtrA_fw	CGGTGGGGCATTCTAGAATCTTGTTTGGTGGTTTGCGCAG
29_ES_RF_2g17430_pSK275_rev	GCCTAGATGGCCTCTTGCATCTTGCATCCGTTCTGGGCCG

Supplemental Table S1. Primers used in this study to generate *A. fumigatus* mutant strains

1_ES_ 4g07940_1_fw	GTCACCTGCAGGCTTGATCT
4_ES_ 4g07940_2_rev	AATCTCTGCTCCGCGACAAA
31_ES_ 2g09850_1_fw	TCATTGGTGGTGTCGGCTAC
36_ES_ 2g09850_3_rev	CAGCTTGGCAACAGTAGGGA

Supplemental Table S2. Primers used for validation of deletion strains

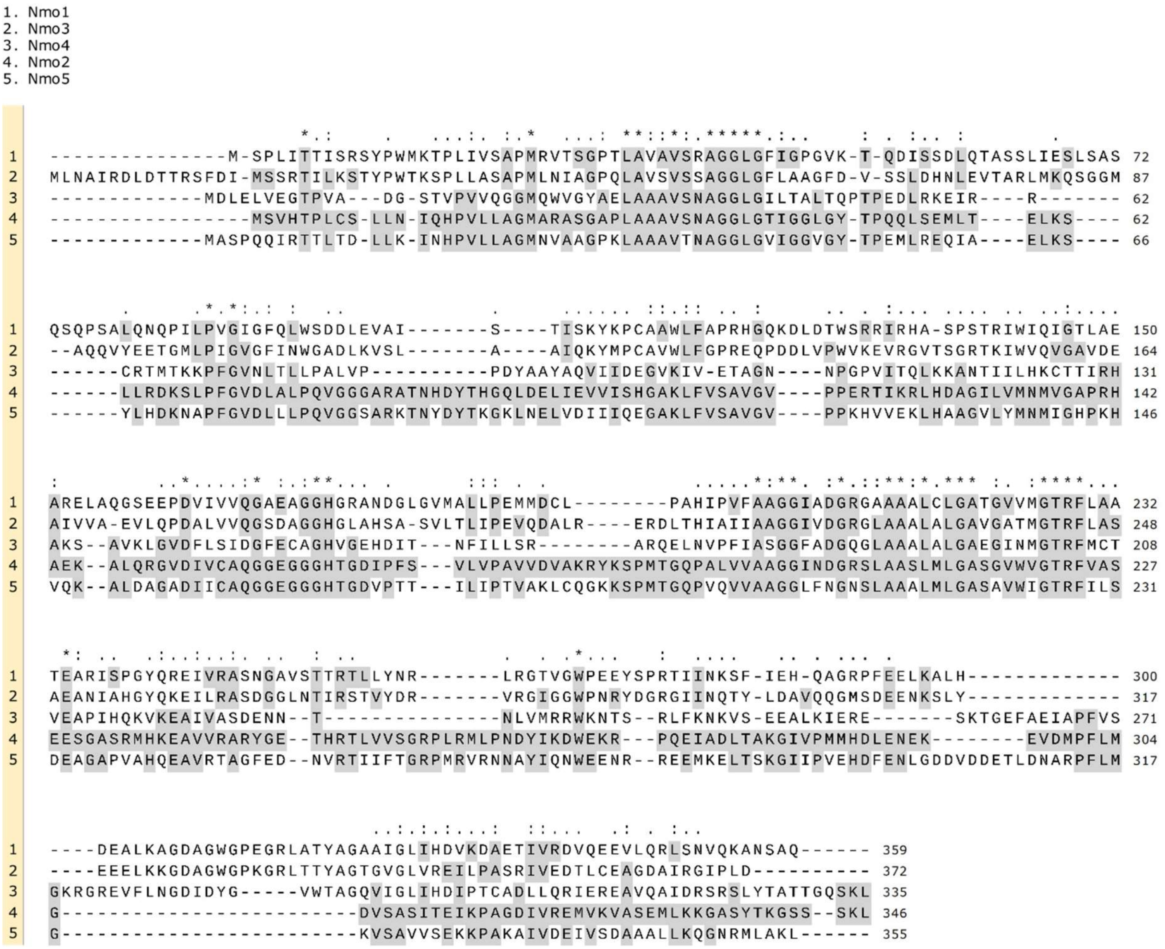

Supplemental Figure S2. Protein sequence analysis of Nmos in *A. fumigatus*

Alignment of Nmo sequences was performed using Clustal Omega and obtained result in FASTA format was submitted to SnapGene. Sequence of Nmo2 was used as a reference sequence. Matching sequences were highlighted in grey

Computational prediction of cellular localization of Nmos

Here, we performed a computational prediction of intracellular localization and potential secretion of Nmos in *A. fumigatus* (Table S1). We used SignalP 4.0 [44] to predict presence of signalling peptides that target proteins to secretory pathways. This analysis showed that none of the Nmos contained a canonical signalling peptide. Two prediction tools, namely SherLoc2 and MultiLoc2 [45,46] predicted that Nmo1, Nmo2, Nmo4, and Nmo5 were peroxisomal, while Nmo3 was predicted to be mitochondrial. Finally, Yloc could predict with a high confidence that Nmo2 was secreted and Nmo4 was localized to peroxisome [47]. To consolidate Yloc's prediction that Nmo2 is secreted with the absence of a signal peptide as determined using SignalP 4.0, OutCyte 1.0

was used in order to determine whether the protein might be secreted via an unconventional protein secretion pathway (UPS) [48]. With a score of 0.513, Nmo2 is classed as an UPS by the tool. Overall, our analysis revealed that Nmo1, Nmo4, and Nmo5 are likely either cytoplasmic or peroxisomal, while Nmo2 likely is secreted via UPS. In addition, Nmo3 was predicted to be mitochondrial.

**Protein**	**Signaling peptide?**	**Predicted Localization**		
		**SherLoc2**	**MultiLoc2**	**YLoc**
**Nmo1**	No	Peroxisome	Peroxisome	Peroxisome (-)
**Nmo2**	No	Peroxisome	Peroxisome	Secreted Pathway (++)
**Nmo3**	No	Mitochondrium	Mitochondrium	Plasma Membrane (-)
**Nmo4**	No	Peroxisome	Peroxisome	Peroxisome (++)
**Nmo5**	No	Peroxisome	Peroxisome	Cytoplasm (-)

Supplemental Table S3. Signal peptide and cellular localization predictions for *A. fumigatus* Nmos.

Signal peptide predictions were made using SignalP 4.0 [44]. SherLoc2 and MultiLoc2 both do not return confidence scores [45,46]. Yloc returns confidence scores both in natural language and with a numerical score [47].

Confirmation of functionality and phylogeny of NMOs found in *Aspergillus fumigatus, Metarhizium brunneum* and *Magnaporthe oryzae*

In order to confirm that both Nmo2 and Nmo5 of *Aspergillus fumigatus* have all necessary protein domains to be fully functional, the protein sequences of *A. fumigatus*, *M. oryzae* and *M. brunneum* were used as query for an rpsblast search (e-value < 1e-5; with parameters -seg no -comp_based_stats [49]) of the Conserved Domain Database (CDD; version 3.19 [50]). This database encompasses NCBI-curated domains and data imported from Pfam, SMART, COG, PRK, and TIGRFAMs. For that purpose, *M. oryzae* sequence were retrieved based on published literature [12] and used as query to find the highest scoring protein in *M. brunneum* using blastp with a cutoff of 1e-5 to find corresponding Nmos in *M. brunneum* [49]. Additional, Interpro Scan (version 5.56 [51]) was used to scan for domains and visualize them (Figure S3).

Table S4 and Figure S3 confirms that all domains necessary for Nmo2 and Nmo5 to function are present and domains found correspond to the ones found in functional *M. oryzae* Nmos [12]. Therefore, canonical functionality of the enzymes can be assumed.

Additionally, Nmo1, Nmo2, Nmo3, Nmo4 and Nmo5 of *M. oryzae*, *A. fumigatus* and *M. brunneum* were aligned using MAFFT (v7.490; --localpair and –maxiterate 1000 options) [52], alignments were assessed using SIAS (http://imed.med.ucm.es/Tools/sias.html) and a maximum likelihood tree was generated using IQ-Tree v1.6.12 [53] with LG+I+G4 as a substitution model, determined using ModelFinder [54]. Branch support was determined using SH-aLRT tests and the tree was midpoint rooted.

Sequence identity and similarity are higher between *M. oryzae* and *M. bruneu* Nmo2 and Nmo5 than between either of them and *Aspergillus fumigatus* (Table S5 and S6). This also reflects in the phylogenetic tree (Figure S4).

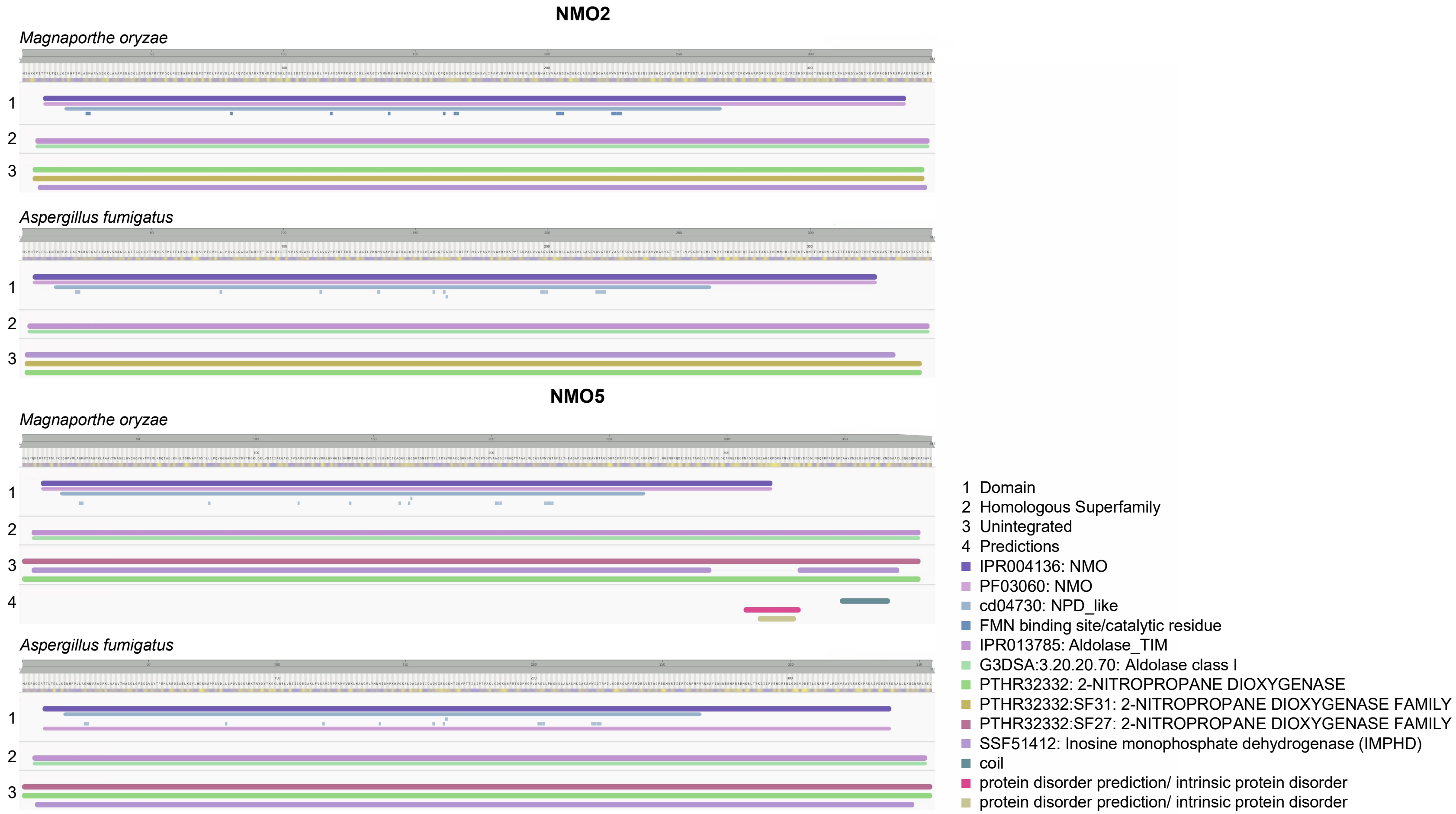

Supplemental Figure S3. Interpro Scan results for Nmo2 and Nmo5 of *Aspergillus fumigatus* and *Magnaporthe oryzae.*

Supplemental Table S4. Conserved protein domains in Nmo2 and Nmo5 of *Magnaporthe oryzae*, *Metarhizium brunneum* and *Aspergillus fumgatus*. Based on scan of CDD profiles (version 3.19) using RPS-BLAST (e-value < 1e-5; with parameters -seg no -comp_based_stats). NPD-like = 2-Nitropropane dioxygenase (NPD); enACPred_II = putative enoyl-[acyl-carrier-protein] reductase II ; NMO = Nitronate monooxygenase; YrpB = NAD(P)H-dependent flavin oxidoreductase YrpB, nitropropane dioxygenase family

**Domain**	**PSSM-id:**	***Magnaporthe oryzae***		***Metarhizium brunneum***		***Aspergillus fumigatus***	
		**Nmo2**	**Nmo5**	**Nmo2**	**Nmo5**	**Nmo2**	**Nmo5**
**NPD_like**	CDD:240081	+	+	+	+	+	+
**YrpB**	CDD:224981	+	+	+	+	+	+
**enACPred_II**	CDD:132195	+	-	-	-	+	-
**NMO**	CDD:367316	+	+	+	+	+	+

**A**			
	Metarhizium brunneum	***Aspergillus fumigatus***	***Magnaporthe oryzae***
Metarhizium brunneum	1	0.61	0.74
Aspergillus fumigatus	0.59	1	0.59
Magnaporthe oryzae	0.73	0.6	1
**B**			
	Magnaporthe oryzae	***Aspergillus fumigatus***	***Metarhizium brunneum***
Magnaporthe oryzae	1	0.76	0.76
Aspergillus fumigatus	0.68	1	0.71
Metarhizium brunneum	0.74	0.77	1

Supplemental Table S5. Global similarity (Blosum62) results of MAFFT alignment of A) Nmo2s and B) Nmo5s in *Magnaporthe oryzae*, *Metarhizium brunneum* and *Aspergillus fumigatus*. Statistics generated using SIAS (http://imed.med.ucm.es/Tools/sias.html) with default parameters (gap opening penalty of 10, gap extension penalty of 0.5)

A

Metarhizium brunneum	100%		
Aspergillus fumigatus	62.71%	100%	
Magnaporthe oryzae	72.83%	63.87%	100%
	Metarhizium brunneum	***Aspergillus fumigatus***	***Magnaporthe oryzae***

B

Magnaporthe oryzae	100%		
Aspergillus fumigatus	75.21%	100%	
Metarhizium brunneum	75.19%	76.05%	100%
	Magnaporthe oryzae	***Aspergillus fumigatus***	***Metarhizium brunneum***

Supplemental Table S6. Identity results of MAFFT alignment of (A) Nmo2s and (B) Nmo5s in *Magnaporthe oryzae*, *Metarhizium brunneum* and *Aspergillus fumigatus*. Statistics generated using SIAS (http://imed.med.ucm.es/Tools/sias.html) using length of smallest sequence as denominator.

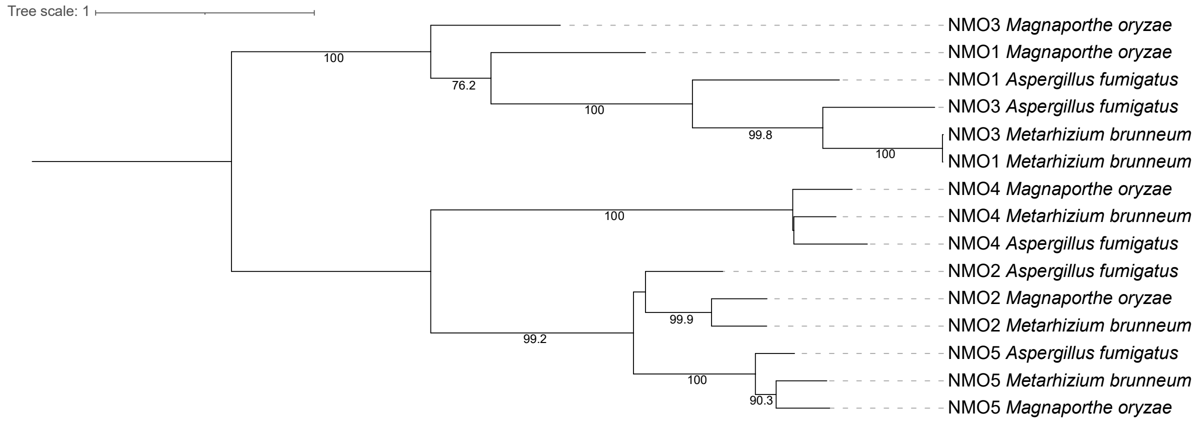

Supplemental Figure S4. Maximum likelihood tree of Nmo1-5 in *Aspergillus fumigatus*, *Magnaporthe oryzae* and *Metarhizium brunneum*. Branch supports indicated were determined using SH-like approximate likelihood ratio tests (SH-aLRT)

Strains	Genotype	Accession number	Selection marker	Reference
CEA10 (CBS144.89)	*A. fumigatus* clinical wild type isolate (WT)	A1163		[55]
Δ*nmo2*	Nitronate monooxygenase 2 (AFUA_4G07940) deletion mutant of CEA10	AFUA_4G07940 (*nmo2* gene)	Pyrithiamine resistance	In this study
Δ*nmo5*	Nitronate monooxygenase 5 (AFUA_2G09850) deletion mutant of CEA10	AFUA_2G09850 (*nmo5* gene)	Pyrithiamine resistance	In this study

Supplemental Table S7. *Aspergillus fumigatus* strains used in this study

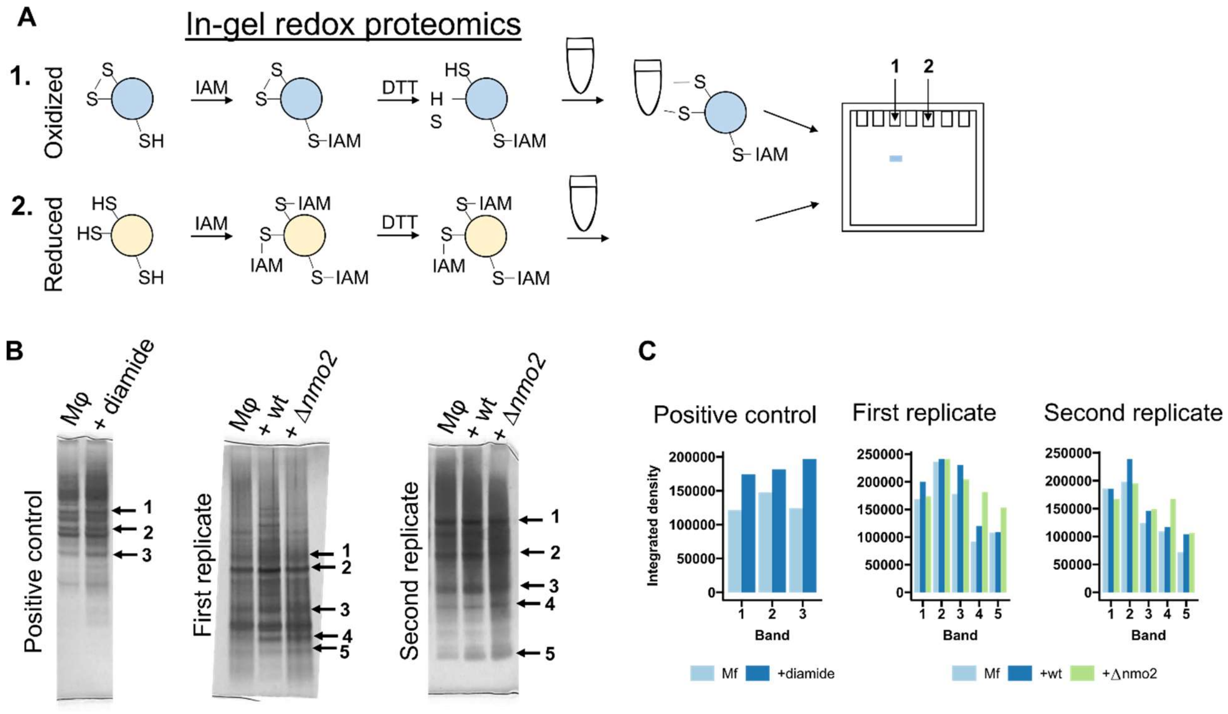

Supplemental Figure S5. Protein oxidation in infected BMDMs. (A). Scheme of the preparation and enrichment method for visualisation of reversibly oxidized proteins. (B). Silver stained SDS gel images of enriched oxidized proteins isolated from infected macrophages. Treatment with 0.5 mM diamide was used as a positive control. (C). Quantification of intensity of corresponding bands on gels as shown on (B)

44. Petersen, T.N.; Brunak, S.; von Heijne, G.; Nielsen, H. SignalP 4.0: Discriminating Signal Peptides from Transmembrane Regions. *Nat. Methods* **2011**, *8*, 785–786. https://doi.org/10.1038/nmeth.1701.
45. Blum, T.; Briesemeister, S.; Kohlbacher, O. MultiLoc2: Integrating Phylogeny and Gene Ontology Terms Improves Subcellular Protein Localization Prediction. *BMC Bioinformatics* **2009**, *10*, 274. https://doi.org/10.1186/1471-2105-10-274.
46. Briesemeister, S.; Blum, T.; Brady, S.; Lam, Y.; Kohlbacher, O.; Shatkay, H. SherLoc2: A High-Accuracy Hybrid Method for Predicting Subcellular Localization of Proteins. *J. Proteome Res.* **2009**, *8*, 5363–5366. https://doi.org/10.1021/pr900665y.
47. Briesemeister, S.; Rahnenfuehrer, J.; Kohlbacher, O. YLoc—an Interpretable Web Server for Predicting Subcellular Localization. *Nucleic Acids Res.* **2010**, *38*, W497–W502. https://doi.org/10.1093/nar/gkq477.
48. Zhao, L.; Poschmann, G.; Waldera-Lupa, D.; Rafiee, N.; Kollmann, M.; Stühler, K. OutCyte: A Novel Tool for Predicting Unconventional Protein Secretion. *Sci. Rep.* **2019**, *9*, 19448. https://doi.org/10.1038/s41598-019-55351-z.
49. Camacho, C.; Coulouris, G.; Avagyan, V.; Ma, N.; Papadopoulos, J.; Bealer, K.; Madden, T.L. BLAST+: Architecture and Applications. *BMC Bioinformatics* **2009**, *10*, 421. https://doi.org/10.1186/1471-2105-10-421.
50. Lu, S.; Wang, J.; Chitsaz, F.; Derbyshire, M.K.; Geer, R.C.; Gonzales, N.R.; Gwadz, M.; Hurwitz, D.I.; Marchler, G.H.; Song, J.S.; et al. CDD/SPARCLE: The Conserved Domain Database in 2020. *Nucleic Acids Res.* **2020**, *48*, D265–D268. https://doi.org/10.1093/nar/gkz991.
51. Blum, M.; Chang, H.-Y.; Chuguransky, S.; Grego, T.; Kandasaamy, S.; Mitchell, A.; Nuka, G.; Paysan-Lafosse, T.; Qureshi, M.; Raj, S.; et al. The InterPro Protein Families and Domains Database: 20 Years On. *Nucleic Acids Res.* **2021**, *49*, D344–D354. https://doi.org/10.1093/nar/gkaa977.
52. Katoh, K.; Standley, D.M. MAFFT Multiple Sequence Alignment Software Version 7: Improvements in Performance and Usability. *Mol. Biol. Evol.* **2013**, *30*, 772–780. https://doi.org/10.1093/molbev/mst010.
53. Nguyen, L.-T.; Schmidt, H.A.; von Haeseler, A.; Minh, B.Q. IQ-TREE: A Fast and Effective Stochastic Algorithm for Estimating Maximum-Likelihood Phylogenies. *Mol. Biol. Evol.* **2015**, *32*, 268–274. https://doi.org/10.1093/molbev/msu300.
54. Kalyaanamoorthy, S.; Minh, B.Q.; Wong, T.K.F.; von Haeseler, A.; Jermiin, L.S. ModelFinder: Fast Model Selection for Accurate Phylogenetic Estimates. *Nat. Methods* **2017**, *14*, 587–589. https://doi.org/10.1038/nmeth.4285.
55. Thau, N.; Monod, M.; Crestani, B.; Rolland, C.; Tronchin, G.; Latgi, J.; Paris, S. Rodletless Mutants of Aspergillus Fumigatus. *Infect. Immun* **1994**, 62, 4380–4388. https://doi.org/10.1128/iai.62.10.4380-4388.1994.
